# Primary Corneal Squamous Cell Carcinoma Confined to the Cornea: An Unusual Presentation of Ocular Surface Squamous Neoplasia Without Limbal Involvement

**DOI:** 10.7759/cureus.83565

**Published:** 2025-05-06

**Authors:** Hideki Fukuoka, Takuya Matsumoto, Yoshihiro Yoshitani, Minori Minamide, Chie Sotozono

**Affiliations:** 1 Department of Ophthalmology, Kyoto Prefectural University of Medicine, Kyoto, JPN

**Keywords:** corneal epithelium, corneal squamous cell carcinoma, epithelial scraping, occupational exposure, ocular surface squamous neoplasia (ossn), primary corneal neoplasia, surgical excision, topical 5-fluorouracil, without limbal involvement

## Abstract

Ocular surface squamous neoplasia manifests primarily on the limbus, with occasional extension onto the cornea. Primary corneal squamous cell carcinoma (SCC) devoid of limbal involvement is an exceptionally rare occurrence. The present case report details a 58-year-old male painter who experienced progressive blurred vision for three months and was diagnosed with SCC strictly confined to the corneal epithelium in his right eye, with no extension to the limbus. Clinical examination revealed significantly reduced visual acuity (0.2 or 20/100 with correction) and diffuse corneal epithelial opacity without limbal or conjunctival involvement, which was initially unresponsive to topical steroid treatment. The patient had no significant medical history. His occupational exposure to painting materials may constitute a potential risk factor. The unique manifestation of SCC, limited to the cornea, posed a significant diagnostic challenge by deviating from the conventional limbal-originating pattern. The patient underwent a biopsy, followed by a complete surgical excision with intraoperative application of 0.04% mitomycin C (MMC), supplemented by postoperative topical 5-fluorouracil (5-FU) therapy. This case underscores the necessity of considering SCC in the differential diagnosis of persistent corneal epithelial lesions, even when the limbus appears uninvolved, and demonstrates an effective treatment approach for this rare entity.

## Introduction

Ocular surface squamous neoplasia (OSSN) signifies a continuum of dysplastic alterations in the ocular surface epithelium, ranging from mild dysplasia to invasive squamous cell carcinoma (SCC) [1]. OSSN is typically classified based on the degree of epithelial involvement (dysplasia, carcinoma in situ, or invasive carcinoma) and the anatomical location (conjunctival, limbal, or corneal with limbal involvement). These lesions have been observed to originate at the limbus, the transition zone between corneal and conjunctival epithelia, where there is a high concentration of limbal stem cells [2]. The conventional presentation of this condition almost invariably involves the limbus, with a vast majority of cases demonstrating limbal involvement. SCC that is isolated to the cornea without limbal involvement is an exceptionally rare condition, with only a few documented cases in the literature [3-5].

Risk factors for OSSN include ultraviolet light exposure, human papillomavirus infection, human immunodeficiency virus, and smoking [6]. The standard treatment approach for OSSN involves surgical excision with safety margins, potentially supplemented with topical chemotherapy to reduce recurrence rates [7,8].

This case report presents an extremely rare variant of OSSN, a primary corneal SCC without any limbal involvement, which falls outside the conventional anatomical classification. We describe its clinical presentation, histopathological findings, and successful management.

## Case presentation

A 58-year-old male patient presented with a three-month history of progressively worsening blurred vision in his right eye. Initial treatment at a local clinic included topical fluorometholone for presumed corneal epithelial inflammation. However, this treatment did not result in the resolution of the symptoms, and visual acuity continued to decline. The patient's occupation, which involved painting work, potentially exposed him to various chemical irritants. The patient reported no significant past medical history and denied smoking.

**Figure 1:**
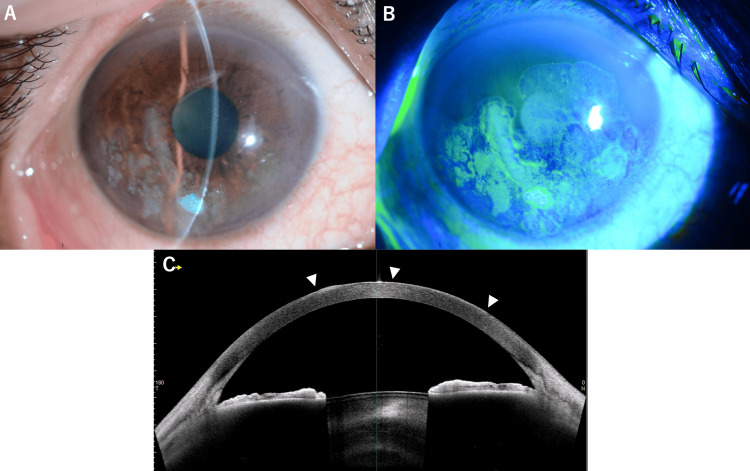
Clinical and imaging findings of the primary corneal squamous cell carcinoma (A) Slit-lamp photograph of the right eye showed a white, elevated lesion confined to the corneal surface without limbal involvement. The lesion had an irregular surface and appears to be limited to the corneal epithelium. (B) Fluorescein staining image demonstrated the corneal lesion and the irregular epithelial surface with areas of hyperkeratosis, confirming the lesion was strictly limited to the cornea with no extension to the limbus. (C) Anterior segment optical coherence tomography (AS-OCT) of the right eye showed corneal epithelial thickening corresponding to the squamous cell carcinoma (white arrowheads). The lesion was confined to the epithelial layer without invasion into the corneal stroma, consistent with the diagnosis of corneal squamous cell carcinoma without limbal involvement.

Upon presentation to our hospital, his best-corrected visual acuity was 0.2 (20/100) in the right eye and 1.2 (20/16) in the left eye. Intraocular pressure measurements were 16.7 mmHg in the right eye and 19.3 mmHg in the left eye. A slit-lamp examination of the right eye revealed a diffuse opacity of the corneal epithelium without obvious limbal involvement. Conjunctival involvement was not observed. The anterior segment was otherwise unremarkable, with a deep and quiet anterior chamber (Figure 1).

**Figure 2:**
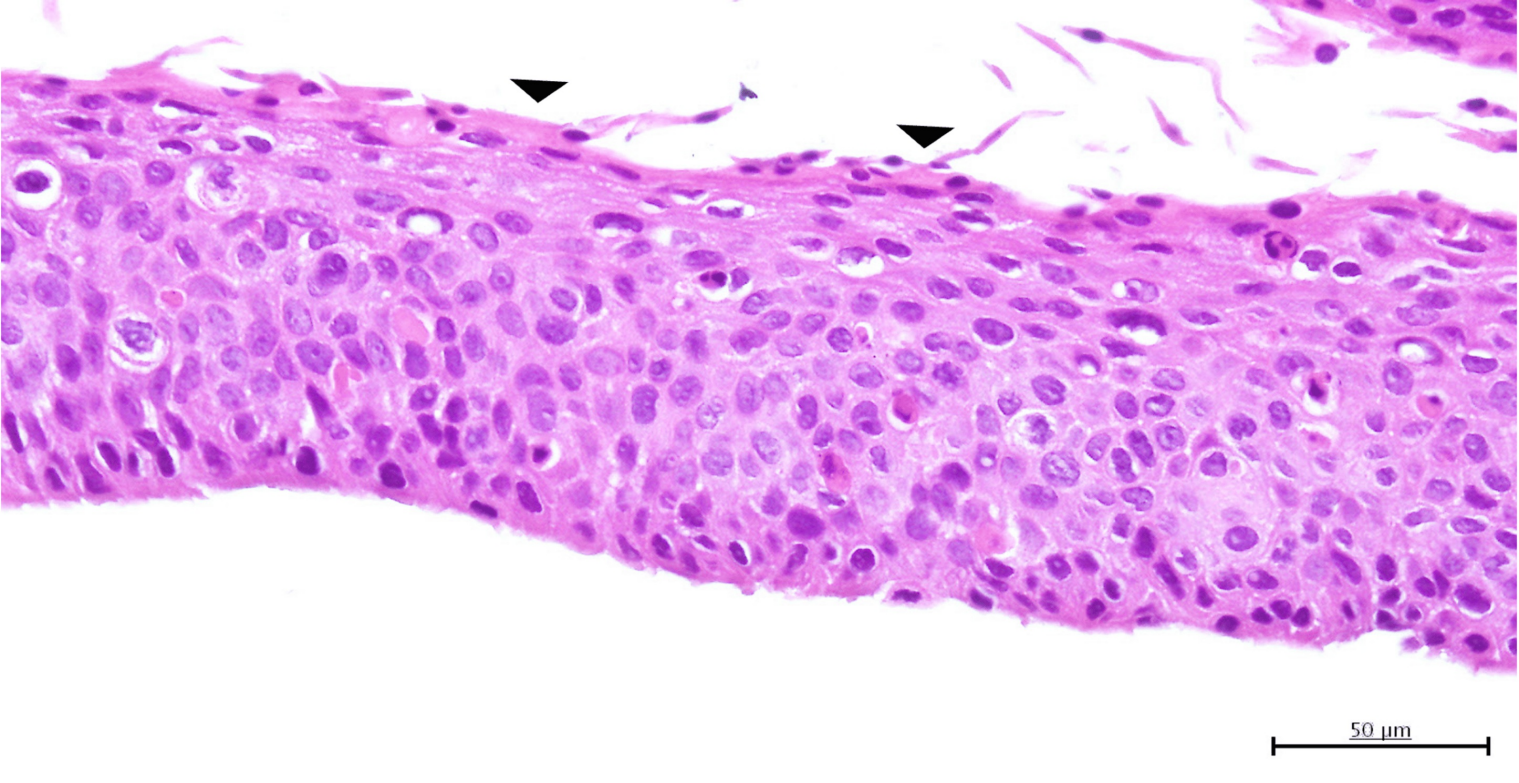
Histopathological image of the corneal squamous cell carcinoma Histopathological analysis using hematoxylin and eosin (H&E) staining revealed replacement of the normal corneal epithelium with atypical squamous epithelial cells, exhibiting thickening, individual cell parakeratosis (black arrowheads), cellular pleomorphism with variation in cell size and shape, and loss of cellular polarity, consistent with squamous cell carcinoma.

The procedure involved epithelial scraping for diagnostic purposes (biopsy) rather than the complete removal of the lesion. Only the superficial epithelial layer was removed to obtain tissue for histopathologic examination while preserving the underlying corneal structure. Histopathological analysis revealed replacement of the normal corneal epithelium with atypical squamous epithelial cells, exhibiting individual cell keratinization and mitotic figures, consistent with SCC (Figure 2).

The specimen was confined to the epithelial tissue, with no stromal invasion.

Following confirmation of the diagnosis, the patient underwent a corneal tumor excision with a 2 mm safety margin, achieving complete removal of all the visible tumoral tissue. After excision, 0.04% mitomycin C (MMC) was applied to the excised margin for three minutes and the area was thoroughly irrigated. A therapeutic soft contact lens was placed at the end of the procedure to promote epithelial healing. One week after the procedure, a course of topical 1% 5-fluorouracil (5-FU) was initiated, administered four times a day for a duration of one week, as an adjuvant therapy to eradicate any potential microscopic disease.

**Figure 3:**
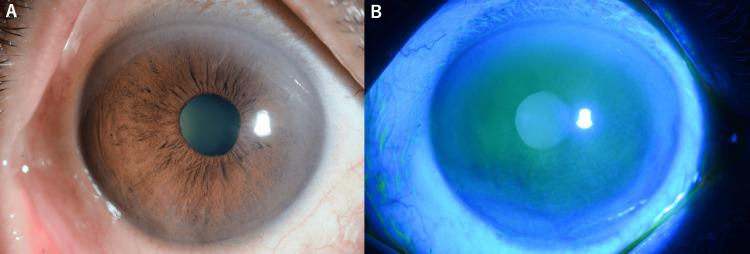
Post-treatment appearance of the right eye (A) Slit-lamp photograph of the right eye following complete surgical excision and topical 5-fluorouracil therapy. The cornea showed complete resolution of the squamous cell carcinoma with restoration of normal corneal clarity. No residual lesion was visible, and the limbus remained uninvolved. (B) Fluorescein staining showed the healed corneal surface. The epithelium appeared intact and regular with normal staining pattern, indicating successful treatment without significant scarring. No evidence of recurrence was observed.

The patient's visual acuity improved to 0.9 (20/22) in the right eye three months after treatment. No recurrence was observed during the follow-up period, and the corneal surface healed well without significant scarring (Figure 3).

## Discussion

This case demonstrated an exceptionally rare manifestation of SCC, characterized by its restriction to the corneal epithelium without involvement of the limbus. The preponderance of OSSN cases arising from the limbus is attributable to the presence of stem cells that are susceptible to malignant transformation, a process that is frequently initiated by exposure to ultraviolet radiation and other carcinogens [9].

The diagnosis of primary corneal SCC without limbal involvement poses significant challenges, as it can mimic non-neoplastic conditions such as infectious keratitis, unresponsive keratitis or corneal opacities [5]. In the present case, the initial diagnosis was misguided, leading to treatment with corticosteroids for a presumed corneal inflammation. This underscores the necessity of maintaining a high level of suspicion for neoplasia in cases of treatment-resistant corneal epithelial lesions.

The morphological classification of OSSN encompasses gelatinous, leukoplakic, papilliform, and velvety types, while growth patterns may manifest as either diffuse or nodular [10]. The diffuse, non-invasive nature of the patient's lesion may explain its limited extent and the good response to treatment.

Surgical excision with clear margins is the gold standard treatment for OSSN [11]. Adjuvant topical chemotherapy with agents such as 5-FU, MMC, or interferon-α2b can help prevent recurrence by eliminating residual microscopic disease [12,13]. The selection of 5-FU for our patient was guided by its favorable efficacy-to-toxicity profile and its limited duration of application. While interferon-α2b is recognized as a less toxic alternative to 5-FU for OSSN treatment, our therapeutic choice was constrained by the Japanese healthcare insurance system, which does not cover interferon-α2b for this indication. The high cost of interferon therapy rendered it inaccessible for our patient, compelling us to utilize 5-FU as a more economically viable option with proven efficacy.

The molecular pathogenesis of corneal intraepithelial neoplasia (CIN), one of OSSNs, involves increased expression of cell proliferation markers such as Ki67 and human telomerase reverse transcriptase (hTERT). Corneal OSSN frequently exhibits increased apoptosis and a lack of epithelial-mesenchymal transition (EMT), which may underlie the generally low metastatic potential of these lesions compared to other SCCs [14].

## Conclusions

Primary SCC that is confined to the cornea is an exceedingly rare clinical entity that can be effectively managed with surgical excision followed by topical chemotherapy. This case underscores the significance of incorporating OSSN in the differential diagnosis of persistent corneal epithelial lesions, even in the absence of limbal involvement. The corneal-limited presentation of SCC poses unique diagnostic challenges because it can mimic various non-neoplastic conditions, leading to delayed diagnosis and treatment.

Our experience demonstrates that a combination approach of complete surgical excision with safety margins, intraoperative MMC application, and postoperative 5-FU therapy provides excellent outcomes for this rare variant of OSSN. Adequate diagnosis and suitable treatment can lead to substantial visual enhancement and mitigate the likelihood of subsequent occurrences or progression. Long-term follow-up is imperative to monitor for potential recurrence, though the prognosis appears favorable when treated appropriately.
